# Progressive Pseudorheumatoid Dysplasia resolved by whole exome sequencing: a novel mutation in WISP3 and review of the literature

**DOI:** 10.1186/s12881-019-0787-x

**Published:** 2019-03-29

**Authors:** Ben Pode-Shakked, Asaf Vivante, Ortal Barel, Shai Padeh, Dina Marek-Yagel, Alvit Veber, Shachar Abudi, Aviva Eliyahu, Irit Tirosh, Shiri Shpilman, Shirlee Shril, Friedhelm Hildebrandt, Mordechai Shohat, Yair Anikster

**Affiliations:** 1 Metabolic Disease Unit, Edmond and Lily Safra Children’s Hospital, Sheba Medical Center, 52621 Tel-Hashomer, Israel; 2 Division of Nephrology, Department of Medicine, Boston Children’s Hospital, Harvard Medical School, Boston, MA 02115 USA; 3 The Dr. Pinchas Borenstein Talpiot Medical Leadership Program, Sheba Medical Center, Tel-Hashomer, Israel; 4 Sheba Cancer Research Center, Sheba Medical Center, Tel-Hashomer, Israel; 5 Pediatric Rheumatology Unit and Department of Pediatrics, Edmond and Lily Safra Children’s Hospital, Sheba Medical Center, Tel-Hashomer, Israel; 6 Sackler Faculty of Medicine, Tel-Aviv University, Tel-Aviv, Israel; 7 The Wohl Institute for Translational Medicine, Sheba Medical Center, Tel-Hashomer, Israel

**Keywords:** WISP3, CCN6, Progressive pseudorheumatoid dysplasia, PPRD, Pseudorheumatoid arthritis of childhood

## Abstract

**Background:**

Progressive pseudorheumatoid dysplasia (PPRD) is a rare autosomal-recessive, non-inflammatory arthropathy, shown to be caused by mutations in the WNT1-inducible signaling pathway protein 3 (*WISP3*) gene. Although several hundred cases were reported worldwide, the diagnosis remains challenging. Subsequently, the syndrome is often unrecognized and misdiagnosed (for instance, as Juvenile Idiopathic Arthritis), leading to unnecessary procedures and treatments. The objective of the current study was to identify the molecular basis in a family with PPRD and describe their phenotype and course of illness.

**Patients and methods:**

We present here a multiply affected consanguineous family of Iraqi-Jewish descent with PPRD. The proband, a 6.5 years old girl, presented with bilateral symmetric bony enlargements of the 1st interphalangeal joints of the hands, without signs of synovitis. Molecular analysis of the family was pursued using Whole Exome Sequencing (WES) and homozygosity mapping.

**Results:**

WES analysis brought to the identification of a novel homozygous missense mutation (c.257G > T, p.C86F) in the *WISP3* gene. Following this diagnosis, an additional 53 years old affected family member was found to harbor the mutation. Two other individuals in the family were reported to have had similar involvement however both had died of unrelated causes.

**Conclusion:**

The reported family underscores the importance of recognition of this unique skeletal dysplasia by clinicians, and especially by pediatric rheumatologists and orthopedic surgeons.

**Electronic supplementary material:**

The online version of this article (10.1186/s12881-019-0787-x) contains supplementary material, which is available to authorized users.

## Background

Progressive pseudorheumatoid dysplasia (PPRD, OMIM 208230), also referred to as pseudorheumatoid arthritis of childhood (PPAC) or spondyloepiphyseal dysplasia tarda with progressive arthropathy (SEDT-PA), is an autosomal recessive disorder, shown to be caused by mutations in the WNT1-inducible signaling pathway protein 3 (*WISP3*) gene, located on chromosome 6q22 [7, 12]. *WISP3* is a member of the CCN family of genes, which encode growth factors with multiple roles in connective tissue, including the regulation of cell proliferation, differentiation and migration [7]. This rare panethnic skeletal dysplasia typically presents with restriction of hip movement, and progressive involvement of the metacarpophalangeal (MCP), proximal (PIP) and distal interphalangeal (DIP) joints, wrists, elbows, knees, shoulders and ankle joints. In large case series, the presenting features were reported to be swelling of the small interphalangeal joints, contractures, fatigability and gait anomalies [3, 6]. While pain and joint swelling occur in the early disease stages, signs of joint inflammation are absent. The disease is typically silent in infancy, and usually manifests between the ages of 3 and 6 years [6]. While normal in infancy, growth usually gradually slows, and the adult height is often well below the 3rd percentile. So far, several hundred cases have been reported in the literature with varied phenotypes, and an array of causative mutations identified throughout the *WISP3* gene [2, 3, 6] (For an extensive list of all *WISP3* variants reported in patients with PPRD to date, see Additional file 1: Table S1). Nevertheless, the diagnosis remains elusive and is often only made years following the onset of symptoms. This can be attributed to several reasons, including the relatively limited joint involvement often characterizing the initial presentation, the nonspecific findings of joint swelling without signs of arthritis, and the fact that the specialist to which the patients present is more often a pediatrician, rheumatologist or orthopedic surgeon, and only rarely a medical geneticist.

## Methods

Research subjects’ blood samples and pedigree data were obtained from individuals with a diagnosis of Progressive pseudorheumatoid dysplasia. Written, informed consent was obtained from the affected individuals or their legal guardians for both genetic analysis and publication of patients’ photographs and imaging studies. Approval for human subject research was obtained from the Institutional Review Boards of the medical centers involved.

### Whole exome sequencing

Whole Exome Sequencing (WES) and variant analysis were performed as previously described [14, 15]. Briefly, Agilent Sure Select human exome capture arrays (Life Technologies™) were performed followed by next generation sequencing (NGS) on an Ilumina™ sequencing platform. CLC Genomics Workbench (version 6.5.1) software (CLC bio) was used to map sequence reads against the human reference genome (NCBI build 37/hg19). Ensuing WES, genetic variants were initially filtered to preserve only non-synonymous changes followed by filtering to maintain only alleles with a Minor Allele Frequency (MAF) of < 0.01. In order to estimate for MAF, an integrated datasets containing all available data from the Genome Aggregation Database (gnomAD), the Exome Variant Server (EVS) project, dbSNP142, and the 1000 Genomes Project was used. Next, we analyzed observed sequence variants for the presence of paralogous genes, pseudogenes, or misalignments via the UCSC Human Genome Bioinformatics Browser. We then examined all variants within the sequence alignments of the CLC Genomic Workbench™ software program for mismatches that indicate potential false alignments and for poor sequence quality. Finally, we assessed variants for evolutionary conservation using web-based programs to determine whether these variants represent known disease-causing mutations and to predict the effect of disease candidate variants on the encoded protein. Under an assumed autosomal recessive mode of inheritance, variant calling was carried out by cell biologists and/or geneticists at both Sheba Medical Center and Boston Children’s Hospital, who had access to the clinical phenotypes, family pedigrees, homozygosity mapping results and WES analyses, according to proposed guidelines [9]. For the sequence variants that remained, these were further evaluated for segregation in affected and unaffected individuals in the family. Following this process for all variants within the homozygous regions, a single candidate variant remained.

### Homozygosity mapping

Using the CLC Genomics Workbench™ (version 6.5.2) software (CLC bio, Aarhus, Denmark), sequence reads were mapped to the human reference genome assembly (NCBI build 37/hg19),

Files were created via CLC Genomics Workbench™ (version 6.5.2) software (CLC bio, Aarhus, Denmark) and BAM. Picard and samtools [8] was used for downstream processing of aligned BAM files. GATK [13] was used to perform SNV calling and the generated VCF file was subsequently used in homozygosity mapper [11].

### Detection of mutation frequency in 100 Iraqi-Jewish controls via restriction assays

DNA was extracted using Magnapure Automatic machine from five milliliters of heparinized blood that were drawn from each subject. DNA amplification was performed in a 25 μl reaction containing 50 ng of DNA, 10uM of each and Red load Taq Master*5 (LAEOVA). An initial denaturation of 5 min at 95 °C was followed by 30 cycles (94 °C for 30 s, 60 °C for 30 s, and 72 °C for 30 s) and then by final extension at 72 °C for 10 min. Sequencing was performed using an automated ABI Prism 3100 Genetic Analyzer (Perkin Elmer).

Confirmation of mutations and carrier rates were performed via restriction assays. The sequence that included the c.257G > T mutation was amplified via the following primers: 5’CCTGTTTGGGGGAAATCTTCT3′ -F and 5’TCCAAGCTAACAATTGCAGGAA 3’ –R, resulting in 459 bp PCR product. BsrD1 restriction enzyme that cuts the normal alleles, was used in order to cut the products, yielding 160 bp and 299 bp products.

## Results

### Clinical characteristics

#### Patient V:1

**Fig. 1:**
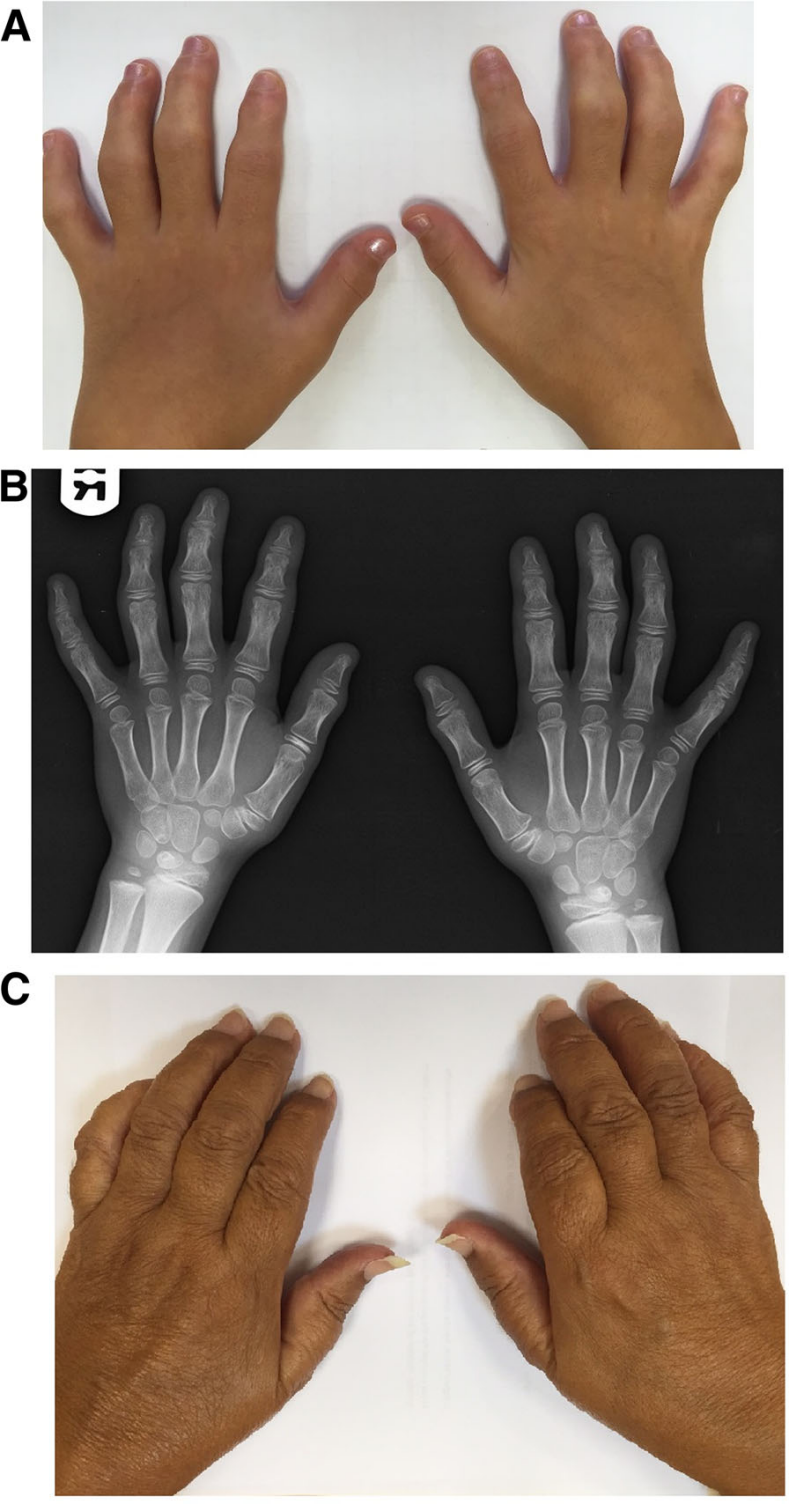
Clinical and radiographic findings in Progressive Pseudorheumatoid Dysplasia. **a** Bilateral symmetric bony enlargements of the 1st interphalangeal joints of patient V:1 (at the age 9.5 years). **b** Radiograph of patient V:1 (at age 6.5 years) demonstrating interphalangeal joint metaphyseal enlargement. **c** Camptodactyly with reduced range of motion of multiple hand joints, and bilateral bony enlargement of the interphalangeal joints of patient III:13 (at the age of 53 years)

The proband (designated patient V:1) is a 6.5 years old girl, born to consanguineous parents of Iraqi Jewish descent, who first presented to the pediatric rheumatology clinic with bilateral painless thickening of PIPs joints of two months duration. She denied any history of fever, trauma or rash. She was born after uneventful pregnancy and labor, at a birth weight of 3700 g. Her previous medical history was notable only for several episodes of pneumonia before the age of 3 years, and normocytic anemia. On presentation, physical examination revealed symmetric thickening of the DIPs and PIPs of both hands with no evidence of joint effusion, tenderness or stress pain. All joints showed normal range of motion. There was no evidence for tenosynovitis. All other joints showed no signs of synovitis, tenosynovitis or enthesitis. Muscle strength was normal and no evidence for muscle wasting. The skin was clear and no nail changes were noted. Her height was 110 cm (10th percentile) and weight was approximately 19 kg (~30th percentile). The initial evaluation included radiographs of the hands demonstrating short distal phalanges with bilateral swelling of soft tissue around the DIPs and PIPs (Fig. 1b); normal radiographs of the feet and pelvis; radiograph of the spine which showed shortening of the intervertebral spaces D 4–5-6 and D 10–11-12; and laboratory tests which included a complete blood count with absolute and relative eosinophilia (2.2 K/microL, Normal < 0.7 K/microL; 24.2%), normal C-reactive protein (CRP) and erythrocyte sedimentation rate (0.07 mg/l and 13 mm/hour, respectively), and biochemistry, CPK, complement levels, Rheumatic factor (RF) and serum lipid profile all within normal range. Testing for antinuclear antibody (ANA) was negative as well. Ophthalmological evaluation was normal and showed no evidence of uveitis, and abdominal ultrasound showed no evidence of hepatosplenomegaly. During several hospitalizations to our center an extensive evaluation ensued, and several working diagnoses were entertained, including mucopolysaccharidosis, pachydermatodactyly and Winchester syndrome.

On a follow up visit at the age of 9.5 years, she had a further progression of the joint deformation (Fig. 1a), including restricted range of motion of the neck (80° on extension, 60° on lateral rotation bilaterally) and wrists, limited flexion and extension of the fingers at the PIPs level, knees and ankles. Back movements were within normal range. Once again, no evidence of active synovitis, tenosynovitis or enthesitis was noted. Due to the multiple joint involvement, physical therapy, occupational therapy and hydrotherapy were initiated in order to maintain range of motion.

#### Patient III:13

**Fig. 2:**
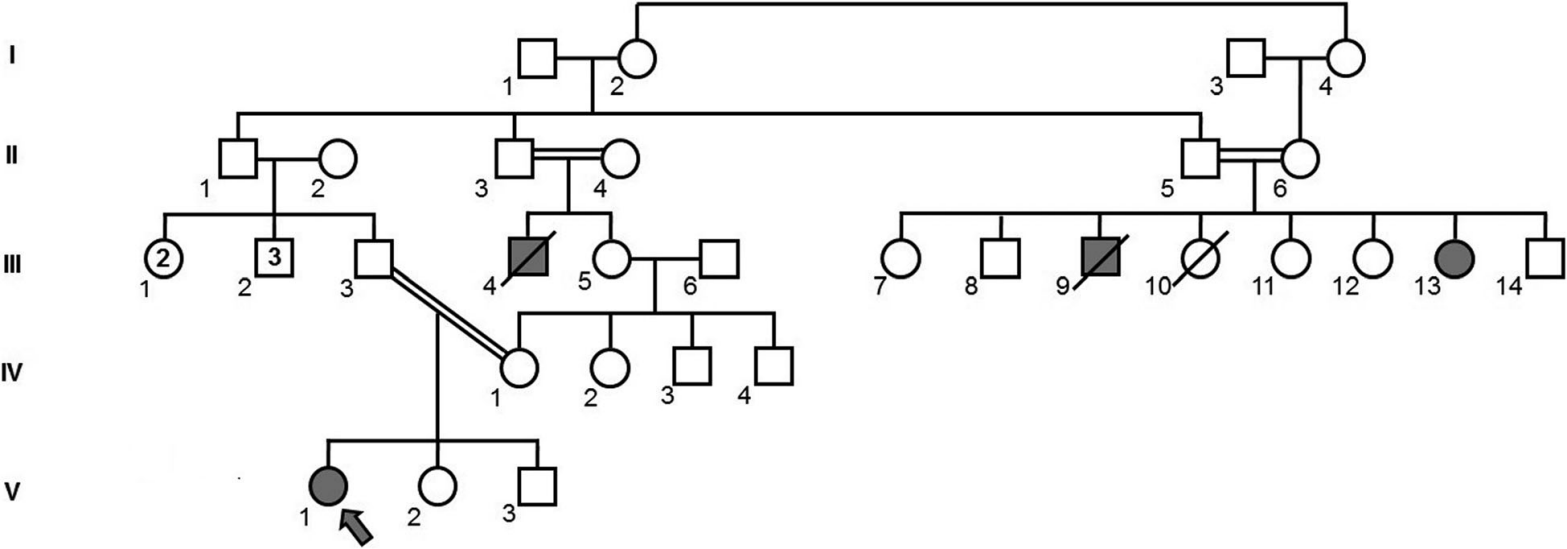
Genogram of a multiply affected family with Progressive Pseudorheumatoid Dysplasia. Proband is denoted by the arrow. Full symbols designate affected individuals. Sequencing of the *WISP3* gene revealed the affected individuals (V:1, III:13) to be homozygous for a novel c.257G > T mutation

Following meticulous family history, obtained for the proband, three additional relatives were reported to be similarly affected (Fig. 2). These included the proband’s late maternal uncle (patient III:4), as well as two siblings, cousins to the proband’s father: a 53 years old woman (patient III:13), and her late brother (III:9).

Patient III:13 was seen at our center at the age of 53 years. She was the 7th child of consanguineous parents, and had reported multiple joint deformations beginning at early childhood, without arthritis. She had undergone several surgical interventions, including bilateral hip replacement at the age of 28 years, bilateral knee replacement at the age of 45 years, and disc nucleoplasty at the age of 52 years. She complained of difficulties standing and walking for significant distances, and for several years had begun the intermittent use of a wheelchair. She had also noted infrequent lower back pain which tends to increase throughout the day. Upon examination, short stature (130 cm) and bilateral swelling around the DIPs and PIPs were noted (Fig. 1c). Additional active medical problems including decreased sight acuity in her left eye and hypertension, for which she had been treated with antihypertensive agents. Treatment with Pamidronate (Aredia) was also initiated by a rheumatologist one year prior.

Patient III:4 was noted to have similar physical appearance as the other affected family members, with short stature and multiple joint involvement, and had died at the fifth decade of life following complications of heart disease.

Patient III:9 was reported to have died at the age of 18 years, following accidental suffocation, however he was noted to have short stature (approximately 135 cm) and similar joint involvement as his affected sister.

### Molecular analysis- whole exome sequencing

In order to reach a molecular diagnosis in the family, DNA was extracted from whole blood samples from the proband, her parents and her father’s affected aunt. Whole Exome Sequencing was performed, and had led to the identification of the previously unreported c.257G > T (p.C86F) missense mutation on exon 3 of the *WISP3* gene (NM_003880.3), for which both affected individuals were homozygous and both of the proband’s parents were heterozygous. The affected amino acid residue was found to be highly conserved throughout evolution, and pathogenicity prediction software (including PolyPhen2, MutationTaster, FATHMM and SIFT, among others) predicted the mutation to be pathogenic.

### Mutation frequency analysis in a cohort of healthy controls of Iraqi-Jewish descent

Carrier frequency screening for the c.257G > T mutation in DNA samples from 107 healthy controls of Iraqi-Jewish descent (214 alleles) revealed a single asymptomatic carrier (MAF (minor allele frequency) = 0.0046), corresponding with a roughly estimated prevalence of over 1:40,000 homozygotes in this population.

## Discussion

Using a positional-candidate strategy, Hurvitz et al. first described mutations in *WISP3*, a member of the CCN family of genes, as the molecular basis of PPRD [7]. Since then, nearly seventy loss-of-function variants in *WISP3*, also known as *CCN6*, have been reported thus far in several hundreds of individuals with PPRD of diverse ethnic origins, including frameshifts, deletions and missense mutations [4, 6, 10] (Additional file 1: Table S1). These did not show clear genotype-phenotype correlations.

Mutation analysis in the kindred reported herein revealed both affected individuals available for analysis to be homozygous for the previously unreported c.257G > T mutation. These results enable planning of future pregnancies and genetic counseling in the extended family. As this family is the first reported affected family of Iraqi-Jewish descent, mutation carrier frequency was pursued and found to correspond with an estimated prevalence of over 1:40,000 homozygotes in this population. Of note, in cases in which Whole Exome Sequencing fails to identify the disease-causing mutation, it has been suggested that intronic mutations with subsequent splicing aberrations can be detected in cDNA extracted from fibroblasts [6]. In the case at hand, WES could have been avoided, and a more targeted approach (i.e. single gene sequencing, gene panel sequencing) would probably have identified the pathogenic variant, if the putative diagnosis of PPRD would have been reached clinically. Nonetheless, the diagnosis of PPRD is often elusive, as occurred with the family described herein, and hence WES was pursued. Indeed, the advantage of WES over other methodologies is in circumstances in which the specific diagnosis remains unclear, or when the differential diagnosis for a proband or family still includes several relevant genes.

On a clinical note, due to the relative rarity of the disease, patients with PPRD are often reported to be either misdiagnosed or diagnosed late in the course of the disease. Patient III:13, for instance, first diagnosed at the age of 53 years, demonstrates the typical and significant delay in diagnosis from the first onset of symptoms, as previously mentioned [6]. Often, PPRD is initially misdiagnosed as Juvenile Idiopathic Arthritis (JIA), due to the interphalangeal joints widening, among other common manifestations. However, it is worthy to note that in PPRD, in contrast to JIA, joint synovitis will be missing, serum inflammation markers typically will not be elevated, serum RF serology will be negative, and use of anti-rheumatic agents will not yield clinical benefit [1]. Distinctive radiographic features of PPRD which may also assist in differentiating it from JIA and additional alternative diagnoses include spondyloepiphyseal dysplasia with platyspondyly as an early finding, and the lack of destructive joint erosions [5].

To conclude, we present here a family with multiple affected individuals diagnosed using NGS to harbor a homozygous mutation in *WISP3*, bringing to a molecular diagnosis of PPRD. As early identification and diagnosis of PPRD can prevent unnecessary invasive tests, as well as administration of varied agents including anti-inflammatory drugs and immunosuppressant, it requires a high index of suspicion. Mutations in *WISP3* should therefore be sought in appropriate clinical settings, such as early onset arthritic changes in children, in the absence of elevated inflammation markers, or in the presence of positive family history suggestive of an autosomal recessive disorder. Special recognition of this unique non-inflammatory arthropathy is needed by pediatric rheumatologists and orthopedic surgeons, who are often the specialists to which these patients present. Finally, our data adds to the currently known mutational profile of *WISP3*-associated PPRD.

## Additional file

Additional file 1: **Table S1.** Summary of all previously reported mutations in *WISP3* in patients with Progressive pseudorheumatoid dysplasia (Adapted from Madhuri et al. 2016 and updated). (DOCX 30 kb)

## Abbreviations

ANA: Antinuclear antibody
CRP: C-reactive protein
JIA: Juvenile idiopathic arthritis
MAF: Minor allele frequency
MCP: Metacarpophalangeal, proximal (PIP) and distal interphalangeal (DIP
NGS: Next generation sequencing
PPAC: Pseudorheumatoid arthritis of childhood
PPRD: Progressive pseudorheumatoid dysplasia
RF: Rheumatic factor
SEDT-PA: Spondyloepiphyseal dysplasia tarda with progressive arthropathy
WES: Whole Exome Sequencing
WISP3: WNT1-inducible signaling pathway protein 3
