# Supplementary material for: Progressive Pseudorheumatoid Dysplasia resolved by whole exome sequencing: a novel mutation in WISP3 and review of the literature
Source: BMC Med Genet. 2019 Mar 29;20:53. doi: 10.1186/s12881-019-0787-x (PMC6439983; doi:10.1186/s12881-019-0787-x)
Supplement: Supplementary file 1 — Table S1. Summary of all previously reported mutations in WISP3 in patients with Progressive pseudorheumatoid dysplasia (Adapted from Madhuri et al. 2016 and updated). (DOCX 30 kb) [file 12881_2019_787_MOESM1_ESM.docx]

**Supplementary Table 1**

Summary of all previously reported mutations in *WISP3* in patients with Progressive pseudorheumatoid dysplasia (Adapted from Madhuri et al. 2016 and updated).

| Exon/Intron | Nucleotide change | Amino acid change | Ethnic origin | Reference |
| --- | --- | --- | --- | --- |
| Exon 1 | c.43delGC | p.A15fs* | United States | Hurvitz 1999 |
| Exon 1 | 9kb deletion encompassing exon 1 and the 5′UTR | | Morocco | Neerinckx 2015 |
| Intron 1 | g.insT+2IVS1 | Alters intron 1 splice donor site | Jordan | Hurvitz 1999 |
| Intron 1 | c.49-1G>A |  | India | Bhavani 2015 |
| Exon 2 | c.105dupT | p.G36fs*10 | China | Liu 2015 |
| Exon 2 | c.136C>T | p.G46* | China | Yue 2009;  Yu 2015 |
| Exon 2 | c.156C>A | p.C52* | Turkey, Lebanon, Syria, Italy, France, India, Iran | Hurvitz 1999; Delague 2005; Temiz 2011; Dalal 2012; ; Madhuri 2016; Rai 2016; Sailani 2017 |
| Exon 2 | c.182G>T | p.C61F | Poland | Garcia Segarra 2012 |
| Exon 2 | c.185delC | p.P62Lfs*21 | Turkey | Garcia Segarra 2012 |
| Exon 2 | c.190G>A | p.G64R | Ecuador | Montané 2016 |
| Exon 2 | c.197G>A | p.S66N | United States, Italy, Ecuador | Garcia Segarra 2012; Montané 2016 |
| Exon 2 | c.232T>C | p.C78R | France | Delague 2005 |
| Exon 2 | c.233G>A | p.C78Y | India | Dalal 2012; Ekbote 2013; Madhuri 2016 |
| Exon 2 | c.236-237CC>AA | p.A79E | Italy | Garcia Segarra 2012 |
| Exon 2 | c.246delA | p.P82fs*21 | Saudi Arabia, Jordan | Delague 2005 |
| Exon 2 | c.248G>A | p.G83E | Lebanon, Syria, Turkey, India | Delague 2005; Temiz 2011; Dalal 2012; Ekbote 2013; Madhuri 2016 |
| Exon 2 | c.257G>T | p.C86F | Iraqi-Jewish (Israel) | Our report |
| Exon 2 | c.296A>T | p.Y99F | India | Bhavani 2015 |
| Exon 2 | c.298T>A | p.C100S | India | Bhavani 2015 |
| Exon 2 | c.327C>A | p.Y109* | Turkey | Garcia Segarra 2012 |
| Exon 2 | c.340T>C | p.C114R | India | Dalal 2012 |
| Exon 2 | c.341G>A | p.C114Y | China | Yue 2009 |
| Exon 2 | c.342T>G | p.C114W | China | Sun 2012; Ye 2012;  Yu 2015; Liu 2015 |
| Exon 2 | c.342_343delTG | p.A115Ifs*16 | Turkey | Garcia Segarra 2012 |
| Intron 2 | c.374-2A>G |  | India | Bhavani 2015 |
| Intron 2 | c.347-1_347-3delCAG |  | India | Bhavani 2015 |
| Exon 2/3 | c.348C>A | p.Y116* | India | Dalal 2012; Madhuri 2016 |
| Exon 3 | c.396T>G | p.C132W | China | Yan 2016 |
| Exon 3 | c.433T>C | p.C145R | India | Dalal 2012 |
| Exon 3 | c.434G>A | p.C145Y | Italy | Delague 2005 |
| Exon 3 | c.530C>A | p.S177* | India | Bhavani 2015 |
| Exon 3 | c.535_536delTG or c.536_537delGT | p.C179fs* | Syria | Delague 2005 |
| Exon 3 | c.589G>C | p.A197Lfs*35 | Syria | Delague 2005 |
| Exon 3 | c.589G>A | p.A197Lfs*35 | Italy | Garcia Segarra 2012 |
| Exon 4 | c.593_597delATAGA | p.Y198* | India | Madhuri 2016 |
| Exon 4 | c.621_622delAAinsT | p.K207Nfs*25 | United States | Garcia Segarra 2012 |
| Exon 4 | c.624delA | p.K208fs*24 | China | Liu 2015 |
| Exon 4 | c.624_625insA | p.C209Mfs*21 | China | Ye 2010 |
| Exon 4 | c.643+1G>A |  | India | Rai 2016 |
| Exon 4 | c.667T>G | p.C223G | China | Ye 2012; Luo 2015;  Yu 2015 |
| Exon 4 | c.670dupA |  | China | Hu 2017 |
| Exon 4 | c.670G>A | p.G224R | Italy | Garcia Segarra 2012 |
| Exon 4 | c.677G>T | p.G226V | United Kingdom | Garcia Segarra 2012; Madhuri 2016 |
| Exon 4 | c.679dupA | p.(C227Lfs*21 | China | Yan 2016 |
| Exon 4 | c.682T>C | p.S228P | India | Dalal 2012; Ekbote 2013 |
| Exon 4 | c.683_684insT | p.N229* | India | Bhavani 2015 |
| Exon 4 | c.685_686insATCTA | p.R230Lfs*4 | India | Bhavani 2015 |
| Exon 4 | c.729_735delGAGAAA | p.M243fs*15 | China | Ye 2010 |
| Exon 4 | c.739_740delTG | p.C247Lfs*31 | Germany, India, Pakistan | Ehl 2004; Dalal 2012; Cassa 2016 |
| Exon 4 | c.740_741delGT | p.C247Lfs*31 | India | Bhavani 2015 |
| Exon 4 | c.756C>A | p.C252X | China | Garcia Segarra 2012; Hu 2017 |
| Exon 4 and intron 4 | c.779_783+1delTAAAGG |  | India | Bhavani 2015 |
| Exon 5 | c.802T>G | p.C268G | India | Dalal 2012 |
| Exon 5 | c.804delC | p.Q269Nfs*44 | India | Bhavani 2015 |
| Exon 5 | c.840delT | p.F280Lfs*33 | China | Zhou 2007 |
| Exon 5 | c.850G>T | p.G284* | Turkey | Garcia Segarra 2012 |
| Exon 5 | c.857C>G | p.S286* | Turkey, China | Garcia Segarra 2012; Yu 2015 |
| Exon 5 | c.863insAC | p.Q289Lfs*25 | United States | Delague 2005 |
| Exon 5 | c.866_867insA (c.866dupA) | p.S290Efs*13 | China | Ye 2010; Sun 2012; Yu 2015 |
| Exon 5 | c.866_867delAG | p.S290Lfs*12 | Iran, Yemen | Delague 2005; Alwabathani 2017 |
| Exon 5 | c.947_951delAATTT | p.Q316Rfs*5 | India | Dalal 2012 |
| Exon 5 | c.993G>A | p.W331* | Italy | Delague 2005 |
| Exon 5 | c.1000T>C | p.S334P | China | Zhou 2007; Sun 2012 |
| Exon 5 | c.1004G>A | p.C335Y | Italy | Garcia Segarra 2012 |
| Exon 5 | c.1010G>A | p.C337Y | India | Dalal 2012; Ekbote 2013; Madhuri 2016 |
| Exon 5 | c.1013A>T | p.Q338L | Japan | Nakamura 2007 |

References

Alawbathani S, Kawalia A, Karakaya M, et al. Late diagnosis of a truncation WISP3 mutation results in a severe phenotype of progressive pseudorheumatoid dysplasia. Cold Spring Harb Mol Care Stud 2017 [Epub ahead of print]; doi:10.1101/mcs.a002139.

Bhavani GS, Shah H, Dalal AB, et al. Novel and recurrent mutations in WISP3 and an atypical phenotype. Am J Med Genet A 2015;167A:2481-2484.

Cassa CA, Smith SE, Docken W, et al. An argument for early genomic sequencing in atypical cases: a WISP3 variant leads to diagnosis of progressive pseudorheumatoid arthropathy of childhood. Rheumatology (Oxford) 2016;55(3):586-9.

Dalal A, Bhavani G SL, Togarrati PP, et al. Analysis of the WISP3 gene in Indian families with progressive pseudorheumatoid dysplasia. Am J Genet A 2012;158A(11):2820-8.

[Delague V](https://www.ncbi.nlm.nih.gov/pubmed/?term=Delague%20V%5BAuthor%5D&cauthor=true&cauthor_uid=16152649), [Chouery E](https://www.ncbi.nlm.nih.gov/pubmed/?term=Chouery%20E%5BAuthor%5D&cauthor=true&cauthor_uid=16152649), [Corbani S](https://www.ncbi.nlm.nih.gov/pubmed/?term=Corbani%20S%5BAuthor%5D&cauthor=true&cauthor_uid=16152649), et al. Molecular study of WISP3 in nine families originating from the Middle-East and presenting with progressive pseudorheumatoid dysplasia: identification of two novel mutations, and description of a founder effect. [Am J Med Genet A.](https://www.ncbi.nlm.nih.gov/pubmed/16152649) 2005 Oct 1;138A(2):118-26.

Ehl S, Uhl M, Berner R, et al. Clinical, radiographic, and genetic diagnosis of progressive pseudorheumatoid dysplasia in a patient with severe polyarthropathy. Rheumatol Int 2004;24:53-6.

[Ekbote AV](https://www.ncbi.nlm.nih.gov/pubmed/?term=Ekbote%20AV%5BAuthor%5D&cauthor=true&cauthor_uid=23270760), [Danda D](https://www.ncbi.nlm.nih.gov/pubmed/?term=Danda%20D%5BAuthor%5D&cauthor=true&cauthor_uid=23270760), [Kumar S](https://www.ncbi.nlm.nih.gov/pubmed/?term=Kumar%20S%5BAuthor%5D&cauthor=true&cauthor_uid=23270760), et al. A descriptive analysis of 14 cases of progressive-psuedorheumatoid-arthropathy of childhood from south India: review of literature in comparison with juvenile idiopathic arthritis. [Semin Arthritis Rheum.](https://www.ncbi.nlm.nih.gov/pubmed/23270760) 2013;42(6):582-9.

Garcia Segarra N, Mittaz L,Campos-Xavier AB, et al. The diagnostic challenge of progressive pseudorheumatoid dysplasia (PPRD): a review of clinical features, radiographic features, and WISP3 mutations in 63 affected individuals. Am J Med Genet C Semin Med Genet 2012;160C:217-29.

Hu Q, Liu J, Wang Y, et al. Delayed-onset of progressive pseudorheumatoid dysplasia in a Chinese adult with a novel compound WISP3 mutation: a case report. BMC Medical Genetics 2017;18(1):149.

Hurvitz JR, Suwairi WM, Van Hul W, et al. Mutations in the CCN gene family member WISP3 cause progressive pseudorheumatoid dysplasia. Nat Genet 1999;23:94-94.

[Liu L](https://www.ncbi.nlm.nih.gov/pubmed/?term=Liu%20L%5BAuthor%5D&cauthor=true&cauthor_uid=25553839), [Li N](https://www.ncbi.nlm.nih.gov/pubmed/?term=Li%20N%5BAuthor%5D&cauthor=true&cauthor_uid=25553839), [Zhao Z](https://www.ncbi.nlm.nih.gov/pubmed/?term=Zhao%20Z%5BAuthor%5D&cauthor=true&cauthor_uid=25553839), [Li W](https://www.ncbi.nlm.nih.gov/pubmed/?term=Li%20W%5BAuthor%5D&cauthor=true&cauthor_uid=25553839), [Xia W](https://www.ncbi.nlm.nih.gov/pubmed/?term=Xia%20W%5BAuthor%5D&cauthor=true&cauthor_uid=25553839). Novel WISP3 mutations causing spondyloepiphyseal dysplasia tarda with progressive arthropathy in two unrelated Chinese families. [Joint Bone Spine.](https://www.ncbi.nlm.nih.gov/pubmed/25553839) 2015 Mar;82(2):125-8.

Luo H, Shi C, Mao C, et al. A novel compound WISP3 mutation in a Chinese family with progressive pseudorheumatoid dysplasia. Gene 2015;564(1):35-8.

Madhuri V, Santhanam M, Rajagopal K, et al. WISP3 mutational analysis in Indian patients diagnosed with progressive pseudorheumatoid dysplasia and report of a novel mutation at p.Y198. Bone Joint Res. 2016;5(7):301-6.

[Montané LS](https://www.ncbi.nlm.nih.gov/pubmed/?term=Montan%C3%A9%20LS%5BAuthor%5D&cauthor=true&cauthor_uid=26991965), [Marín OR](https://www.ncbi.nlm.nih.gov/pubmed/?term=%22Mar%C3%ADn%20OR%22%5BAuthor%5D&cauthor=true&cauthor_uid=26991965), [Rivera-Pedroza CI](https://www.ncbi.nlm.nih.gov/pubmed/?term=Rivera-Pedroza%20CI%5BAuthor%5D&cauthor=true&cauthor_uid=26991965), et al. Early severe scoliosis in a patient with atypical progressive pseudorheumatoid dysplasia (PPD): Identification of two WISP3 mutations, one previously unreported. [Am J Med Genet A.](https://www.ncbi.nlm.nih.gov/pubmed/26991965) 2016;170(6):1595-9.

Nakamura Y, Weidinger G, Linag JO, et al. The CCN family member Wisp3, mutant in progressive pseudorheumatoid dysplasia, modulates BMP and Wnt signaling. J Clin Invest 2007;117:3075-86.

[Neerinckx B](https://www.ncbi.nlm.nih.gov/pubmed/?term=Neerinckx%20B%5BAuthor%5D&cauthor=true&cauthor_uid=27081554), [Thues C](https://www.ncbi.nlm.nih.gov/pubmed/?term=Thues%20C%5BAuthor%5D&cauthor=true&cauthor_uid=27081554), [Wouters C](https://www.ncbi.nlm.nih.gov/pubmed/?term=Wouters%20C%5BAuthor%5D&cauthor=true&cauthor_uid=27081554), et al. A homozygous deletion of exon 1 in WISP3 causes progressive pseudorheumatoid dysplasia in two siblings. [Hum Genome Var.](https://www.ncbi.nlm.nih.gov/pubmed/27081554) 2015;2:15049.

Rai E, Mahajan A, Kumar P, et al. Whole exome sequencing identifies novel and recurrent WISP3 mutations causing Progressive Pseudorheumatoid Dysplasia in Jammu and Kashmir-India. Sci Rep 2016;6:27684.

Sailani MR, Chappel J, Inlora J, et al. WISP3 mutation associated with Pseudorheumatoid Dysplasia. Cold Sprin Harb Mol Case Stud 2017 [Epub ahead of print]. doi: 10.1101/mcs.a001990.

[Sun J](https://www.ncbi.nlm.nih.gov/pubmed/?term=Sun%20J%5BAuthor%5D&cauthor=true&cauthor_uid=22685593), [Xia W](https://www.ncbi.nlm.nih.gov/pubmed/?term=Xia%20W%5BAuthor%5D&cauthor=true&cauthor_uid=22685593), [He S](https://www.ncbi.nlm.nih.gov/pubmed/?term=He%20S%5BAuthor%5D&cauthor=true&cauthor_uid=22685593), et al. Novel and recurrent mutations of WISP3 in two Chinese families with progressive pseudorheumatoid dysplasia. [PLoS One.](https://www.ncbi.nlm.nih.gov/pubmed/22685593) 2012;7(6):e38643.

Temiz F, Ozbek MN, Kotan D, et al. A homozygous recurring mutation in WISP3 causing progressive pseudorheumatoid arthropathy. J Pediatr Endocrinol Metab 2011;24:105-8.

Yan W, Dai J, Xu Z, et al. Novel WISP3 mutations causing progressive pseudorheumatoid dysplasia in two Chinese families. Hum Genome Var. 2016;3:16041.

Ye J, Zhang HW, Wang T, et al. Clinical diagnosis and WISP3 gene mutation analysis for progressive pseudorheumatoid dysplasia. Zhonghua Er Ke Za Zhi 2010;48:194-8. (In Chinese).

[Ye J](https://www.ncbi.nlm.nih.gov/pubmed/?term=Ye%20J%5BAuthor%5D&cauthor=true&cauthor_uid=21993478), [Zhang HW](https://www.ncbi.nlm.nih.gov/pubmed/?term=Zhang%20HW%5BAuthor%5D&cauthor=true&cauthor_uid=21993478), [Qiu WJ](https://www.ncbi.nlm.nih.gov/pubmed/?term=Qiu%20WJ%5BAuthor%5D&cauthor=true&cauthor_uid=21993478), et al. Patients with progressive pseudorheumatoid dysplasia: from clinical diagnosis to molecular studies. [Mol Med Rep.](https://www.ncbi.nlm.nih.gov/pubmed/21993478) 2012;5(1):190-5.

[Yu Y](https://www.ncbi.nlm.nih.gov/pubmed/?term=Yu%20Y%5BAuthor%5D&cauthor=true&cauthor_uid=25738435), [Hu M](https://www.ncbi.nlm.nih.gov/pubmed/?term=Hu%20M%5BAuthor%5D&cauthor=true&cauthor_uid=25738435), [Xing X](https://www.ncbi.nlm.nih.gov/pubmed/?term=Xing%20X%5BAuthor%5D&cauthor=true&cauthor_uid=25738435), et al. Identification of a mutation in the WISP3 gene in three unrelated families with progressive pseudorheumatoid dysplasia. [Mol Med Rep.](https://www.ncbi.nlm.nih.gov/pubmed/25738435) 2015;12(1):419-25.

[Yue H](https://www.ncbi.nlm.nih.gov/pubmed/?term=Yue%20H%5BAuthor%5D&cauthor=true&cauthor_uid=19064006), [Zhang ZL](https://www.ncbi.nlm.nih.gov/pubmed/?term=Zhang%20ZL%5BAuthor%5D&cauthor=true&cauthor_uid=19064006), [He JW](https://www.ncbi.nlm.nih.gov/pubmed/?term=He%20JW%5BAuthor%5D&cauthor=true&cauthor_uid=19064006). Identification of novel mutations in WISP3 gene in two unrelated Chinese families with progressive pseudorheumatoid dysplasia. [Bone.](https://www.ncbi.nlm.nih.gov/pubmed/19064006) 2009;44(4):547-54.

Zhou HD, Bu YH, Peng YQ, et al. Cellular and molecular responses in progressive pseudorheumatoid dysplasia articular cartilage associated with compound heterozygous WISP3 gene mutation. J Mol Med (Berl). 2007;85(9):985-96.
